# The benefits of ventriculoperitoneal shunting in normal pressure hydrocephalus patients—a follow-up of three years

**DOI:** 10.1007/s00701-024-06263-z

**Published:** 2024-09-17

**Authors:** Aylin H. Gencer, Frank P. Schwarm, Jasmin Nagl, Eberhard Uhl, Malgorzata A. Kolodziej

**Affiliations:** 1 Department of Neurosurgery, Justus-Liebig-University, Klinikstraße 33, 35392 Gießen, Germany; 2 Department of Neurosurgery, University Hospital, LMU Munich, Marchioninistraße 15, 81377 Munich, Germany

**Keywords:** Normal pressure hydrocephalus, Ventriculoperitoneal shunt, Evans index, Callosal angle, Comorbidity, Valve pressure setting

## Abstract

**Objective:**

The ventriculoperitoneal shunt (VPS) is an established approach in treating normal pressure hydrocephalus (NPH). This study aims to examine the long-term effects of VPS regarding clinical and radiological outcomes, to explore interdependencies with comorbidities and medication, and to determine a suitable opening pressure of the programmable valve.

**Methods:**

127 patients with VPS were retrospectively evaluated. The Hakim triad along with Evans index (EI) and callosal angle (CA) were examined preoperatively and postoperatively at various time points up to over thirty-six months. Preexisting comorbidities and medication were considered. Adjustments to valve settings were documented along with symptom development and complications. Wilcoxon and paired-sample t-tests were used to analyze postoperative change. Chi-square, Eta-squared, and Pearson coefficients were used in correlation analyses.

**Results:**

Relief from individual symptoms was most prominent within the first 6 months (*p* < 0.01). EI and CA significantly decreased and increased, respectively (*p* < 0.05). Postoperative clinical and radiological improvement was largely maintained over the follow-up period. Diabetes mellitus and apoplexy correlated with surgical outcomes (*p* < 0.05). The median opening pressure as a function of overall symptom management was determined to be 120 mmH_2_O for women and 140 mmH_2_O for men.

**Conclusion:**

VPS is effective in treating NPH with respect to both clinical and radiological outcomes, although these two components are independent of each other. Improvement is most pronounced in short-term and maintained in the long-term. Comorbidities have significant influence on the course of NPH. The valve setting does not forecast change in radiological findings; consequently, priority should be placed on the patient’s clinical condition.

## Introduction

Normal pressure hydrocephalus (NPH) is associated with dilatation of cerebrospinal fluid (CSF) spaces manifesting in a constellation of gait disturbance, cognitive impairment, and urinary incontinence (Hakim-Triad). NPH is a chronic progressive disease in which symptoms may manifest asynchronously or incompletely [6, 38]. Amongst the various pathophysiological explanations lies a decrease in elastic compliance of both the cerebral blood vessels and ventricular walls due to advanced age, which lead to periventricular edema build up and restricted blood perfusion to regions responsible for gait, cognitive functions, and continence [10].

Because of the high average age associated with NPH, many patients present with a variety of preexisting comorbidities and medications, which may have an impact on the overall course of the condition. To date only a small number of previous studies have examined the relationship between comorbidities and NPH progression [1, 5, 36].

Radiological indexes are often used for quantitative assessment of the enlargement of internal CSF spaces. The Evans index (EI) is commonplace in detecting ventriculomegaly [27, 37, 46]. An EI value of above 0.3 is indicative hydrocephalus. Likewise, the callosal angle (CA) holds significance for the diagnostic workup [16, 22]. An angle less than 90° can mark the presence of NPH. Imaging is already of great relevance for diagnosis and may additionally be useful in assessing surgical outcomes during follow-up examinations.

The implantation of a ventriculoperitoneal shunt (VPS) has been established as an effective treatment method over several decades [3, 4, 11, 13, 23, 29, 40, 43]. Modern shunt systems include programmable valves, which allow the amount of CSF drained to be easily regulated by adjusting the pressure at which the valve opens. In this regard, the correct choice of valve setting and careful long-term follow-up for early detection of shunt-associated complications represent important cornerstones in NPH treatment.

The aim of this study was to investigate the effects of VPS surgery on the clinical and radiological findings in the long-term, to find possible associations between comorbidities and treatment outcomes, and to determine an optimal opening pressure for the programmable valve in relation to gender, at which maximal management of symptoms with minimal shunt-associated complications is to be expected.

## Material and methods

### Patient cohort

The patient collective for this retrospective, monocentric study was compiled in the Department of Neurosurgery at the University Hospital Giessen. The study was approved by the institution’s research ethics board (03/2020, AZ 206/19).

All patients who had been diagnosed with NPH and surgically treated with a VP shunt between January 2004 and March 2020 were included. Seven out of the 134 patients identified were excluded from study for the following reasons: three patients were treated in an external hospital such that the date of initial surgery was unclear; two patients had no follow-up data available; and two patient were treated with a shunt that was not ventriculoperitoneal. Comprehensive data was thus available for 127 patients.

### Clinical data

The relevant patient data was collected utilizing the available surgical and medical reports. The preoperative presence of gait disturbance, cognitive impairment, and urinary incontinence was binarily recorded. The presentation of the Hakim-Triad was also recorded for the following postoperative time points: within 6 months, at 6 months, at 12 months, at 24 months, at 36 months, and at over 36 months.

The following comorbidities were taken into account: diabetes mellitus, arterial hypertension, peripheral vascular disease, coronary artery disease, heart failure, cardiac arrhythmias, coagulation disorders, polyneuropathy, renal disease, apoplexy, and neurogenic or neurodegenerative diseases (inter alia Parkinson’s and Alzheimer’s disease). Patient records were also reviewed to determine whether an anticoagulant, antidepressant, and/or antiepileptic was included in the medication at discharge.

### Radiological data

**Fig. 1:**
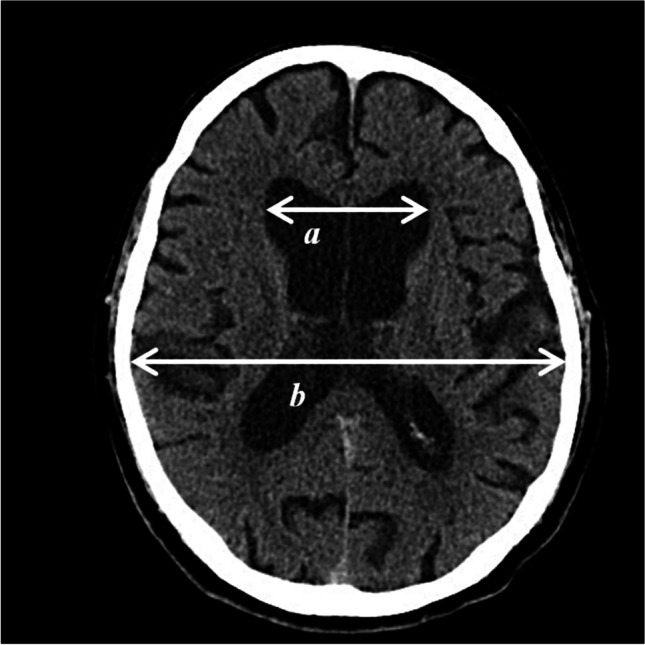
Measurement of Evans index. The maximum width of the anterior horns of the lateral ventricles (**a**), the maximum inner diameter of the skull (**b**). EI = $\frac{a}{b}$

EI is the ratio of the maximum width of the anterior horns of the lateral ventricles (a) to the maximum inner diameter of the skull (b) on the same axial cross-section of CT or MRI images (Fig. 1). EI was calculated for preoperative images and further measurements for follow-up examinations were then conducted at postoperative time points corresponding to the clinical assessment time points.

**Fig. 2:**
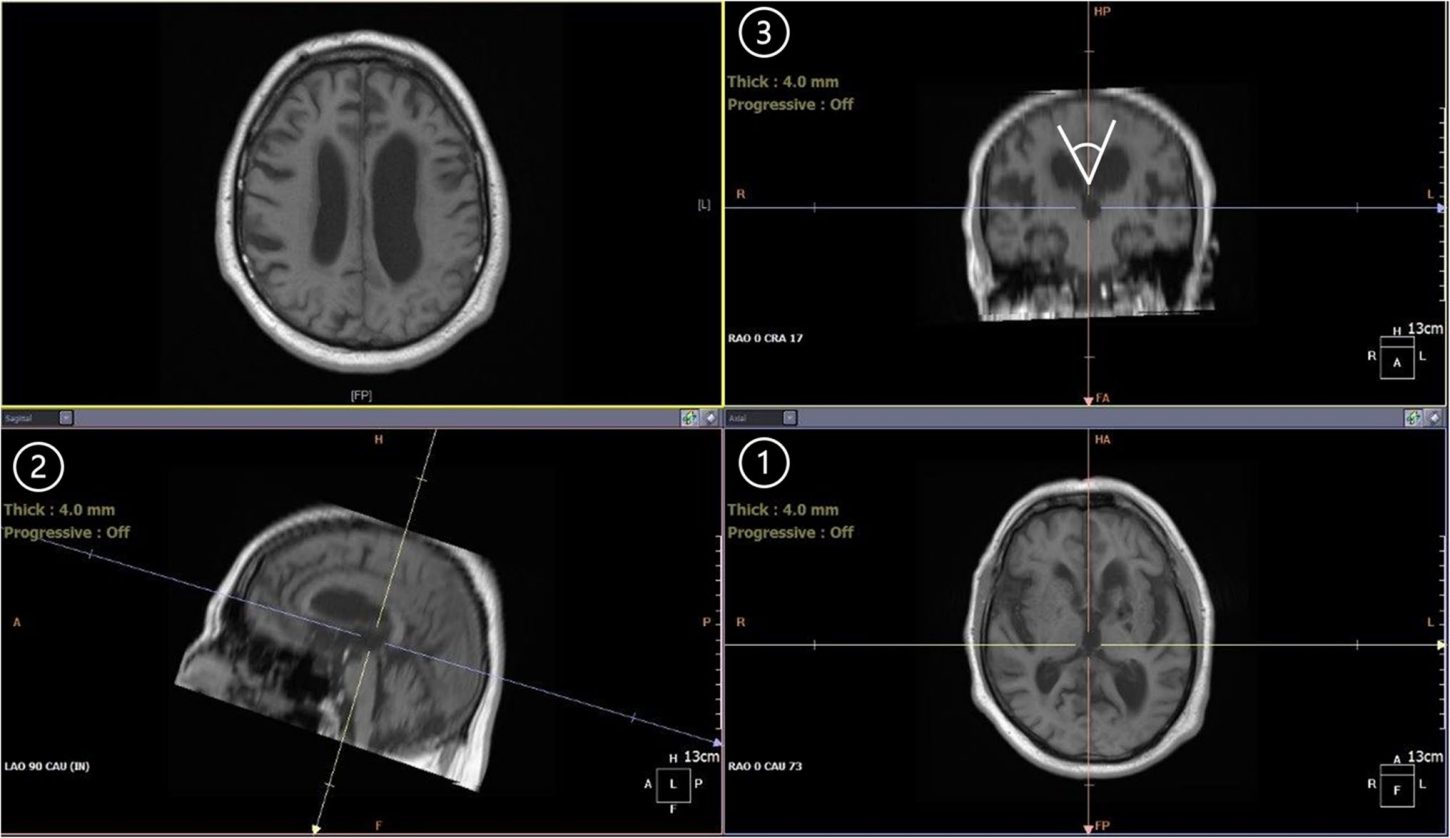
Measurement of the callosal angle. The sagittal orientation line is positioned parallel to the falx cerebri in the axial view (**1**). On the sagittal image, the AC-PC line is identified, and the coronal orientation line is positioned perpendicular to it at the level of the posterior commissure (**2**). On the coronal view, the corpus callosum angle (in white) is measured (**3**)

The callosal angle was measured in the coronal section at the level of the posterior commissure perpendicular to the AC-PC line (anterior commissure—posterior commissure line). This nonstandard angulation required the CT or MRI images to be reconstructed in 3D using the multi-planar reconstruction (MPR) function, in order to compose sagittal and coronal cross-sections from an axial image (Fig. 2). Measurements were again firstly conducted on preoperative images and then on postoperative follow-up images, analogous to the other variables.

### Valve setting

When examining optimal valve settings, only those with Codman® Medos Hakim® shunt systems were included to ensure equivalence. Nine patients, who received an Aesculap Miethke GAV® shunt system, were therefore additionally excluded from valve setting analyses. The presence of the Hakim triad symptoms was ascribed to the corresponding setting for each patient. Similarly, EI and CA values were matched to the complementary valve setting. Shunt-related complications resulting from over- or underdrainage were also recorded for each valve setting. Every adjustment made to the valve setting was attributed to one of the following complications: recurrent or persistent clinical presentation of the Hakim triad, headaches, ventricular size progression, hygroma, shunt dysfunction or revision, occurrence of an additional symptom independent of the Hakim triad, cerebral hemorrhage, imaging findings not further specified, spontaneous readjustment, other/cause unknown.

### Statistical analysis

Statistical analyses were performed using the SPSS statistical program version 28.0 (IBM Corp., Armonk, NY, USA). A two-sided *p*-value < 0.05 was considered statistically significant. Descriptive statistics were presented as absolute and relative frequencies. The mean and standard deviations were calculated for parametric data and median for nonparametric data. EI and CA were analyzed for a difference in means between the preoperative and postoperative states using the paired-sample t-test. To interpret the course of each symptom paired sample Wilcoxon signed-rank test was implemented. Nonparametric correlations were calculated for correlations using the chi-square test (χ^2^). Parametric correlations were examined using the Pearson correlation coefficient (r). A correlation between metric-scaled parameters and nominal-scaled parameters was examined using the Eta squared coefficient (η^2^). Univariate analyses were then performed to determine the significance of the Eta squared coefficient. Multivariate analysis was additionally performed.

## Results

### Demographic

This study included 127 NPH patients (f: 43 (33.9%); m: 84 (66.1%)). The median age at the time of surgery was 74 years (min: 49 years; max: 84 years; interquartile range (IQR) = 10). Patient characteristics are outlined in Table 1. The number of patients with available data on the Hakim triad, EI and CA at each assessment time point is presented in Table 2. The median time of follow-up was 17.3 months.

**Table 1:** Patient characteristics

Attribute	Total number
Cohort	127
Male	84 (66.1%)
Female	43 (33.9%)
^*^Age (in years)	74 (SD: 6.95)
^*^Height (in m)	1.70 (SD: 0.10)
^*^Weight (in kg)	81.80 (SD: 15.22)
^*^BMI	28.49 (SD: 4.74)
*Comorbidities*	
Diabetes mellitus	43 (33.9%)
Arterial hypertension	79 (62.2%)
Peripheral vascular disease	5 (3.9%)
Coronary artery disease	24 (18.9%)
Heart failure	9 (7.1%)
Cardiac arrhythmias	17 (13.4%)
Coagulation disorders	1 (0.8%)
Polyneuropathy	14 (11.0%)
Renal disease	10 (7.9%)
Apoplexy	16 (12.6%)
Neurogenic or neurodegenerative disease	50 (39.4%)
*Medication*	
Anticoagulant	107 (84.3%)
Antidepressant	15 (11.8%)
Antiepileptics	14 (11.0%)

^*^ Mean value; *SD*: standard deviation

**Table 2:** Total number of available data at each time point for clinical and radiological parameters

Parameter	Preop	< 6 Mo Postop	6 Mo Postop	12 Mo Postop	24 Mo Postop	36 Mo Postop	> 36 Mo Postop
Hakim Triad	127	109 (85.8%)	29 (22.8%)	50 (39.4%)	29 (22.8%)	25 (19.7%)	23 (18.1%)
Evans Index	122	88 (72.1%)	38 (31.1%)	42 (34.4%)	28 (23.0%)	28 (23.0%)	31 (25.4%)
Callosal Angle	94	84 (89.4%)	37 (39.4%)	40 (42.6%)	27 (28.7%)	24 (25.5%)	32 (34.0%)

### Clinical outcomes

There was statistically significant negative difference (*p* < 0.05) in every pre- and postoperative pair for the Hakim triad (Table 3). This indicates that the surgical implantation of a VPS achieves reduction of symptoms.

**Table 3:** Outcomes of the Wilcoxon signed-rank test for the Hakim triad comparing the preoperative conditions to those at different postoperative measurement time points

Parameter		< 6 Mo & Preop	6 Mo & Preop	12 Mo & Preop	24 Mo & Preop	36 Mo & Preop	> 36 Mo & Preop
Gait Disturbance	Z	-9.695	-4.243	-5.831	-3.742	-3.742	-2.828
	Sig	< .001^**^	< .001^**^	< .001^**^	< .001^**^	< .001^**^	.005^*^
Cognitive Impairment	Z	-8.832	-4.472	-5.916	-4.583	-3.207	-3.606
	Sig	< .001^**^	< .001^**^	< .001^**^	< .001^**^	.001^**^	< .001^**^
Incontinence	Z	-8.004	-4.000	-5.831	-4.472	-3.771	-3.873
	Sig	< .001^**^	< .001^**^	< .001^**^	< .001^**^	< .001^**^	< .001^**^

^*^ Correlation is significant at the 0.05 level (2-sided)

^**^ Correlation is significant at the 0.01 level (2-sided)

The course of the Hakim triad was further analyzed by comparing only the postoperative data at different time points. For gait disturbance, there was a statistically significant difference when comparing the time point of less than 6 months after surgery with the other postoperative data points (*p* < 0.05). The data show, that although a slight recurrence of gait disturbance was observed after 6 months postoperative, the improvement in gait was largely maintained during the follow up period. Cognitive impairment and incontinence on the other hand, showed no significant difference in postoperative evaluation. This indicates that shunting impacts both cognitive impairment and incontinence, allowing for long-term maintenance of the state of improvement.

**Fig. 3:**
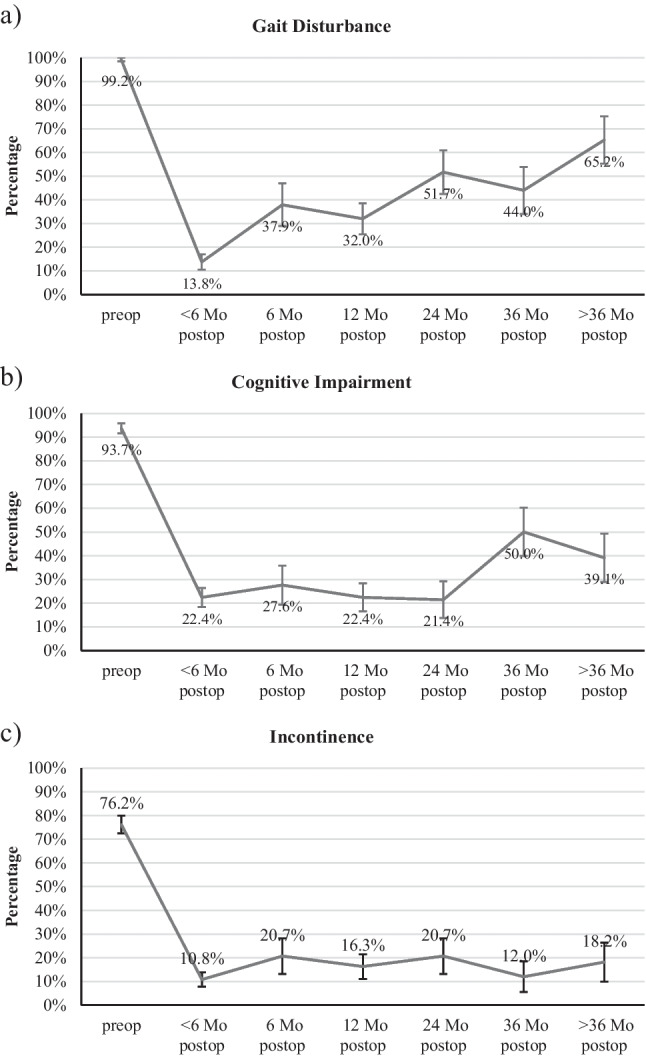
The number of patients with gait disturbance (**a**), cognitive impairment (**b**) and incontinence (**c**) at the different measurement time points (error bars 95%-CI). Paired sample t-test, *p* < 0.05 for all pre- and postoperative pairs

In Fig. 3 the relative frequencies of patients who had gait dysfunction (a), cognitive impairment (b) and incontinence (c) are depicted individually.

The percentage of patients with gait disturbance decreased from 99.2% before surgery to 13.8% shortly after surgery (rate of improvement: 86.1%). Later on, the proportion of patients with gait disturbance slightly increased again but was still lower than preoperative. Cognitive impairment was present in 93.7% of patients preoperatively and was observed in only 22.4% at less than 6 months after surgery (rate of improvement: 76.1%). Incontinence was found in 76.2% of patients prior to VPS but reduced to 10.8% within 6 months following shunting (rate of improvement: 85.8%).

### Radiological outcomes

**Fig. 4:**
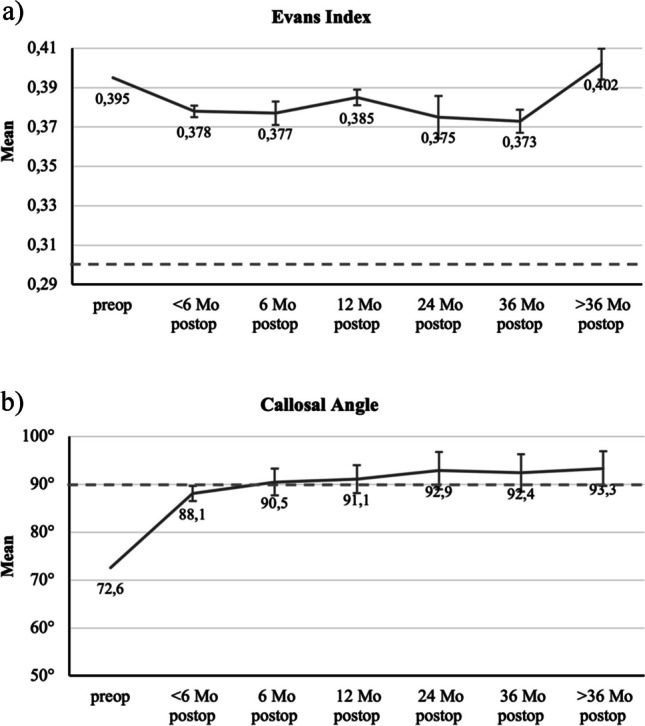
Mean values of the Evans index (**a**) and the callosal angle (**b**) at the different measurement time points (error bars 95%-CI). NPH thresholds marked with dashed line: 0.30 for EI and 90° for CA. Paired sample t-test; EI: *p* < 0.05 for pairs at < 6, 6,12 and 36 months, *p* > 0.05 for pairs at 24 and > 36 months; CA: *p* < 0.05 for all pairs

For both EI and CA, there were significant differences (*p* < 0.05) between the means of preoperative and postoperative measurements (Table 4). EI showed a postoperative decrease indicated by negative t-values, whereas CA increased as demonstrated by positive t-values. The courses of EI and CA throughout the observation period are plotted in Fig. 4.

**Table 4:** Outcomes of the paired-samples t-test for Evans index and callosal angle comparing the preoperative conditions to those at different postoperative measurement time points

Parameter		< 6 Mo & Preop	6 Mo & Preop	12 Mo & Preop	24 Mo & Preop	36 Mo & Preop	> 36 Mo & Preop
Evans Index	T	-5.094	-3.361	-2.632	-1.679	-2.261	-1.02
	Sig	< .001^**^	.002^*^	.012^*^	.106	.032^*^	.316
Callosal Angle	T	8.157	5.970	6.446	4.796	3.309	6.731
	Sig	< .001^**^	< .001^**^	< .001^**^	< .001^**^	.004^*^	< .001^**^

^*^ Correlation is significant at the 0.05 level (2-sided)

^**^ Correlation is significant at the 0.01 level (2-sided)

Although EI significantly decreases postoperatively, on average it does not reach the threshold value of 0.3 representative for non-NPH individuals. At over 36 months after surgery the mean EI (0.402) supersedes that of the preoperative state (0.395). T-test results show a significant difference between under 6 months and over 36 months postoperatively (*p* < 0.05). No further significant difference (*p* > 0.05) was found when postoperative data was compared at different time points. It can therefore be concluded that EI largely retains its stable reduced condition up to 36 months.

In contrast, the mean values of CA not only exhibit a significant improvement but also exceed the threshold of 90° for non-NPH patients starting from the 6-month follow-up assessment. Thereafter, CA progression reaches saturation and remains at a constant over the subsequent months. These findings are supported in t-test analyses of postoperative measurements, as they yielded no significant differences (*p* > 0.05).

### Correlation analysis

A series of statistical correlation analyses were carried out to determine possible associations between all parametric and non-parametric data. A summarized list of statistically significant correlations can be found in Table 5. A pre-existing history of diabetes mellitus positively related to postoperative improvement of both gait disturbance and of CA (*p* < 0.05). Prior history of apoplexy corresponded positively with both the presence of preoperative cognitive impairment (*p* < 0.05) and postoperative improvement in cognitive impairment (*p* < 0.05). Apoplexy also interrelated with higher preoperative values of CA (*p* < 0.05). Increasing age was associated with both greater preoperative cognitive impairment (*p* < 0.05) and higher preoperative EI values (*p* < 0.05). Improvement in EI value was linked positively to premedication with an antidepressant (*p* < 0.05).

**Table 5:** Overview of the significant correlation relationships, the scale of each item, and the statistical tool used

Variable 1 (scale)	Variable 2 (scale)	Test	Value	Sig
Diabetes mellitus (*n*)	Gait improvement (*n*)	χ^2^	.022	.022^*^
Diabetes mellitus (*n*)	CA improvement (*n*)	χ^2^	.028	.028^*^
Apoplexy (*n*)	Cognition preop (*n*)	χ^2^	.029	.029^*^
Apoplexy (*n*)	Cognitive improvement (*n*)	χ^2^	.011	.011^*^
Apoplexy (*n*)	CA preop (m)	η^2^	.156	.024^*^
Age (m)	Cognition preop (*n*)	η^2^	.086	.041^*^
Age (m)	EI preop (m)	r	-.192	.034^*^
Antidepressant (*n*)	EI improvement (*n*)	χ^2^	.017	.017^*^

^*^ Correlation is significant at the 0.05 level (2-sided)

*n*: nominal, m: metric; χ^2^: Chi-square, η^2^: Eta squared coefficient, r: Pearson correlation coefficient

*EI*: Evans index;* CA*: Callosal angle

The strongest parallels were found between EI and CA at each of the corresponding time points (Table 6). The Pearson correlation coefficient of these variables was in all cases negative, meaning that CA increased in value over time, while EI decreased. Highly significant was the correlation between the measurements at the preoperative, less than 6 months and 6 months postoperative time points (*p* < 0.01).

**Table 6:** Assessment for correlations between EI and CA at the different measurement time points. Pearson correlation coefficient (r) was used

	Preop	< 6 Mo Postop	6 Mo Postop	12 Mo Postop	24 Mo Postop	36 Mo Postop	> 36 Mo Postop
Pearson correlation	-.402	-.434	-.524	-.380	-.476	-.152	-.417
Sig. (2-sided)	< .001^**^	< .001^**^	.001^**^	.015^*^	.012^*^	.477	.020^*^
*N*	94	83	35	40	27	24	31

^*^ Correlation is significant at the 0.05 level (2-sided)

^**^ Correlation is significant at the 0.01 level (2-sided)

Remarkably, no statistically significant relationship could be demonstrated between the clinical presentation of symptoms and radiological markers (*p* > 0.05). Although improvement in all variables was demonstrated, the symptoms appear to be independent of the radiological findings and vice versa.

### Valve settings

**Fig. 5:**
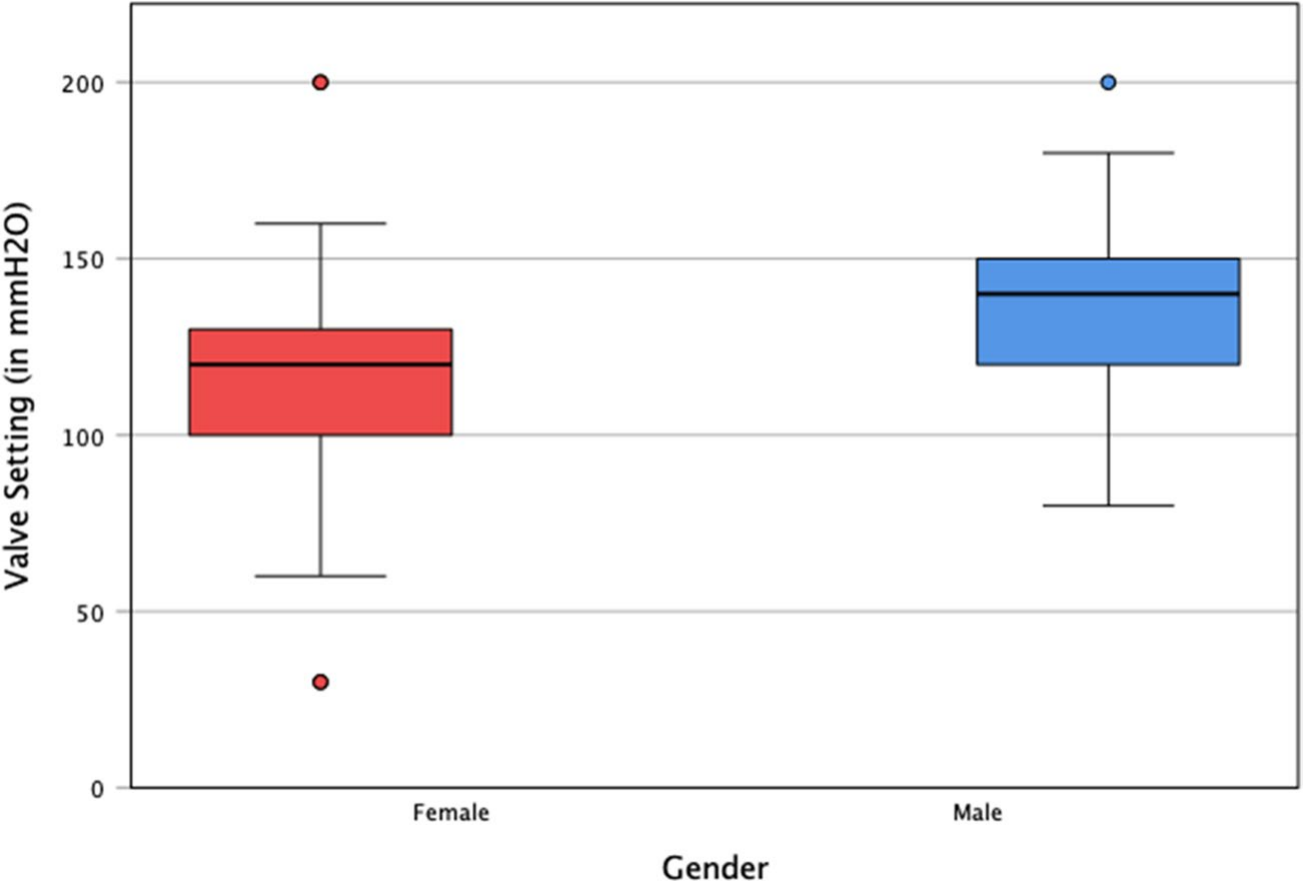
Boxplot of valve setting in mmH_2_O grouped by gender. The median of valve settings (represented by the black lines in the boxes) is 120 mmH_2_O for women (red) and 140 mmH_2_O for men (blue). The medians are plotted with a black line within their respective boxes spanning the first and third quartile. The outliners show the lowest and highest possible settings (30 or 200 mmH_2_O)

The data set of 118 patients (f:43; m:79) outlined above was examined to find valve settings at which none of the Hakim triad symptoms were present. The median valve setting was subsequently calculated dependent on gender. Optimal symptom management was achieved at 120 mmH_2_O for women and at 140 mmH_2_O for men. Figure 5 serves to illustrate these findings as a box plot.

In a large proportion of patients, the valve setting was readjusted after the initial intraoperative setting. A total of 157 readjustments were made in 75 (67%) patients. The reasons for readjustments were manifold and based on complaints described by the patient as well as radiological findings. A summary of all complications can be found in Table 7. One of three possible procedures was implemented in managing each complication: increasing the valve setting, reducing the valve setting or a watch-and-wait approach.

**Table 7:** Complications following shunt implantation and corresponding treatment

Complication	Occurrence	Number of patients	Increase by 10 mmH_2_O	Decrease by 10 mmH_2_O	Watch-and-Wait
Persistence or recurrence of symptoms	63	39 (34.8%)	13 (20.6%)	50 (79.4%)	-
Headaches	34	26 (23.2%)	21 (61.8%)	8 (23.5%)	5 (14.7%)
Progressive ventricle size	6	6 (5.4%)	2 (33.3%)	4 (66.7%)	-
Hygroma	14	14 (12.5%)	11 (78.6%)	2 (14.3%)	1 (7.1%)
Shunt dysfunction or revision	11	10 (8.9%)	4 (36.4%)	4 (36.4%)	3 (27.3%)
Additional symptoms	22	16 (14.3%)	18 (81.8%)	4 (18.2%)	-
Cerebral hemorrhage	7	7 (6.3%)	4 (57.1%)	-	3 (42.9%)
Imaging findings not further specified	5	3 (2.7%)	-	5 (100%)	-
Spontaneous change	6	4 (3.6%)	3 (50%)	2 (33.3%)	1 (16.7%)
Other/unknown cause	27	20 (17.9%)	14 (51.9%)	13 (48.1%)	-

The most observed reason for readjustment was the persistence or recurrence of the Hakim triad, which occurred 63 times in 39 (34.8%) patients. The second most common complaint was headaches, which occurred 34 times in 26 (23.2%) patients.

Potential associations between the valve settings and the radiological markers were investigated. EI correlated negatively and significantly with the lastly selected valve setting for each patient (*p* < 0.05). CA correlated negatively and significantly with the valve setting at which there was maximum symptom management (*p* < 0.05). Regression analysis, however, did not yield any significant results, making causality between the parameters unlikely.

## Discussion

To date, a large number of publications have demonstrated the efficacy of VP shunt implantation in NPH treatment [3, 4, 11, 13, 15, 23, 29, 40, 43]. A common limitation of many of these studies, however, is the relatively short time of follow-up (approximately one year). In this retrospective study, the median follow-up time was 17.3 months, surpassing that of other research groups.

### Effects of VPS on clinical outcomes

In this study, statistically significant reduction of all three symptoms of the Hakim triad was achieved after VPS therapy. Within the first 6 months, gait disturbance showed the most improvement (86.1%), followed by urinary incontinence (85.8%) and cognitive impairment (76.1%). These results are consistent with previous publications demonstrating that VPS is associated with greater control of gait but lesser development of cognitive function [4, 11, 13, 30, 36, 40, 46]. Furthermore, during the three years of follow-up, the number of patients suffering from any of the three symptoms was consistently lower than the preoperative total, demonstrating the longterm benefits of VPS. Prior studies showing preservation of favorable VPS outcomes over five years support these finding [12, 18, 42].

We found that only gait disturbance reemerged slightly as a clinical symptom of NPH during the overall course. This fact, however, could be due to a statistical error caused by the small number of long-term patient data available. On the other hand, gait disturbance is more commonly recognized by patients, which may be a contributing factor in more frequent physician consultations in case of aggravation. This account could also be partly responsible for the higher rate of recurrence of gait dysfunction in our cohort. Further prospective long-term studies are needed to rule out this potential bias.

### Effects of VPS on radiological outcomes

The mean baseline EI in our cohort was 0.395, which reduced significantly to 0.378 within the first six months postoperatively. However, EI never fell below the cut-off value of 0.3. One explanation for this could be that EI is not sensitive enough to detect minute changes in brain morphology after shunting. Alternatively, decreased elasticity that accompanies old age possibly does not allow for continued remodeling towards an entirely normal brain morphology.

The mean baseline CA was 72.6° and increased postoperatively to 88.1° within the first six months. The CA exceeded the cut-off of 90° and remained steadily at normal values in the long-term. These results implicate that CA, in contrast to EI, may be sensitive enough to radiologically project the favorable outcomes of VPS on NPH, suggesting that patients may be able to attain normal brain structure again.

Radiological parameters such as the Evans index and callosal angle are currently used for the most part in the diagnostic workup of NPH [16, 24, 30, 32, 44]. To the best of our knowledge, there are no other studies to date that investigate either EI or CA as follow-up markers for NPH. Our data confirm that both indices undergo postoperative improvement, with EI values decreasing as CA values increase. Further research could verify the suitability of these radiological markers for follow-up examinations.

Curiously, the postoperative course of radiological markers was not reciprocally related to postoperative clinical findings. Within the first 6 months, there was both reduction of symptoms as well as improvement of EI and CA. Even so, gait disturbance returned slightly over the long-term follow-up period, whereas radiological measurements remained largely unchanged. This discrepancy of imaging and clinical findings has often been reported [16, 21, 24, 26, 47]. Consequently, the presentation of the Hakim triad and the patient’s complaints should be the primary factors for assessing the therapeutic success of VPS in daily clinical practice, rather than the radiological findings alone.

### Effects of comorbidities on surgical outcomes

The selection of comorbidities and medications in this study was guided by Wu et al., who similarly examined the impact of VPS on NPH patients [36]. Of particular interest in our study were the vascular comorbidities, which include diabetes mellitus and apoplexy. The underlying rationale is the possible pathophysiological explanation that NPH results from age-induced reduction of blood vessel elasticity and cerebral perfusion. Isrealsson et al. demonstrated that patients with NPH have more vascular risk factors compared to the general population [17].

Wu et al. suggest that a history of diabetes and apoplexy are not significant predictors for clinical VPS outcomes, contrasting the results of our study. We could show a positive correlation of diabetes with a higher rate of improvement of gait disturbance. Noteworthy was the also the association of diabetes with the postoperative increase of CA, resulting in desirable postoperative radiological outcomes. These results in favor of improvement were rather unexpected as diabetes is known to cause oxidative stress on brain tissue and lead to accelerated brain aging [45]. Furthermore, a prior case of apoplexy negatively influences both the preoperative cognitive abilities and the extent of postoperative improvement of these abilities. These results were to be expected as apoplexy is commonly known to lead to cognitive impairment [7, 19, 39]. Another notable finding was that prior apoplexy correlated with increased preoperative CA values. We postulate that this could be due to the fact that apoplexy leads to changes in brain morphology [8, 25], which could in turn cause the CA to pertain higher values despite the presence of NPH.

To our knowledge, no studies to date have explored relationships between comorbidities and radiographic indices, and only two studies (Wu et al. and Nienhaus et al.) have examined associations with the symptoms of the Hakim’s triad. Further trials are therefore needed to verify our findings and to investigate possible explanations for the reported correlations.

### Effects of the valve setting

Currently, there is no consensus on a recommendable opening valve pressures to set initially during surgery. There is a wide range of experience-based studies that differ in opinion regarding an optimal setting. Meier et al. showed that a better clinical outcome was achieved with an initial setting of 50 mmH_2_O compared to settings between 100 and 130 mmH_2_O [41]. In contrast, Bergsneider et al., Larsson et al., and Reinprecht et al. also achieved positive clinical outcomes with a high initial pressure setting [2, 28, 31]. Farahmand et al. investigated the effect of gradually reducing the opening pressure from 200 to 40 mmH_2_O, which however did not yield significantly better outcomes than a static setting of 120 mmH_2_O [9].

In this study, we explored valve settings at which maximum symptom management was obtained. We demonstrated that the median opening pressure, under the condition of complete alleviation of the Hakim triad, was 120 mmH_2_O for women and 140 mmH_2_O for men. These findings are consistent with the recommended pressure levels in the Japanese NPH guidelines for the initial setting [44, 46]. The Quick-Reference-Table (QRT) described in the guidelines considers the patient’s gender, height, and weight. Divergently, we were unable to demonstrate significant association of the valve setting with either height or weight. Further prospective studies are needed to confirm the dependability of these recommended settings in managing symptoms in the long-term.

Shunt-associated complications usually occur due to over-drainage (e.g., subdural hygromas or intracranial hemorrhaging) or under-drainage (e.g., reoccurrence or persistence of clinic). In our cohort, subdural hygromas occurred in a total of 14 (12.5%) cases and intracranial hemorrhaging in 7 (6.3%) cases, following either initial surgery or later valve adjustment. These results are comparable to previous publications. Klinge et al. found that hygromas occurred in 9% and subdural hematomas in 6% [23]. We examined the valve setting in all patients with hygromas, as lower pressure settings could lead to a higher rate of hygromas and found that most of these patients were set to 120 mmH_2_O (an intermediate pressure setting). It should be noted that the majority of patients in our cohort tolerated the opening pressure of 120 mmH_2_O well.

The most common reason for readjustment of the valve setting in our study was the persistence or reoccurrence of symptoms of the Hakim triad. The second most common reason was headache. Larsson et al. found that headaches were common after VPS and that they were more frequent in patients with NPH rather than in the general population [33]. For this reason, long-term follow-up must be mindful of both the serious complications and the mild-to-moderate adverse effects of VPS, as both can greatly affect the patient’s quality of life.

We found that although the radiological parameters EI and CA correlate significantly with the valve setting, no significant results could be obtained in regression analyses. In other words, it cannot be assumed that valve setting is predictive of the course of EI or CA. To our knowledge, this reciprocity or lack thereof has not yet been explored in other studies.

### Limitations

The retrospective design of the study does not allow for standardized documentation throughout the follow-up period. There were no postoperative tests performed in order to quantify symptom improvement (e.g. Timed Up and Go test or Montreal Cognitive Assessment). The different methods in which symptoms are assessed in the follow-up examinations, together with biases by the doctor or the patient, limit the study. Furthermore, the lack of standardized documentation leads to missing radiological and/or clinical data as well as fluctuating postoperative follow-up durations.

While preoperative status could be established in almost all patients, available postoperative data became increasingly sparse in long-term follow-up. Consequently, statistical errors arising from small sample sizes cannot be excluded. In many cases, patients skipped follow-up at certain time points but still presented for later follow-up visits. Patients who are content with the extent of symptom relief tend to contact their doctor less in the long term, which may lead to a selection bias.

Measurements of the CA must be performed on a specific coronary plane that is not standard for CT or MRI. This currently requires the correct angulations and adaptations of the imaging to be done manually by the examiner, which can potentially be a source of inaccuracy. Lee et al. demonstrated that even small malrotations of ≤ 10° can lead to erroneous measurements of CA [35]. In contrast, Park et al. and Virhammar et al. concluded that both CA and EI have high interobserver agreement [20, 24].

Regarding the optimal valve setting, we postulated that patients did not seek further medical care because they were satisfied with symptom control and/or could cope with any adverse side effects of the VPS. However, especially in an elderly patient population, many different reasons could have contributed to a failure to follow-up on the patient’s part, including a waning willingness to travel to the clinic, a change of residence, or even end of life.

## Conclusions

VPS is an effective treatment method for NPH that is well tolerated by patients. Patients benefit most from surgery within the first 6 months, with improvement of gait disturbance being in the forefront. Long-term control of the Hakim triad is largely maintained, demonstrating the pronounced positive impact of VPS treatment. EI and CA show promising results as possible long-term follow-up markers. CA potentially has more value of as a radiologic outcome parameter than EI, as EI is less specific for NPH compared to CA. A history of comorbidities, particularly diabetes mellitus or apoplexy, has significant influence on the course of NPH and should, therefore, be considered during treatment. Desirable equilibrium between maximal symptom management and minimal shunt-associated complications can be maintained at opening valve pressures of 120 mmH_2_O for women and 140 mmH_2_O for men. The valve setting, however, is not predictive for radiographic indices. It is of crucial importance that the patient’s clinical condition be prioritized over radiological findings when establishing an indication for readjustment of the valve pressure setting.

## Data Availability

The study data was collected retrospectively and compiled anonymously. All data collected was in connection with the treatment of patients.
